# Utility of the Collaborative Ocular Tuberculosis Study (COTS) calculator in the management of a case of frosted branch angiitis secondary to presumed intraocular tuberculosis - a case report

**DOI:** 10.1186/s12348-026-00586-x

**Published:** 2026-04-23

**Authors:** Toh Ker Yin, Sowkath Ali, Lekha Gopal, Yuen Yew Sen

**Affiliations:** 1 Department of Ophthalmology, Khoo Teck Puat Hospital, Singapore, Singapore; 2 Department of Ophthalmology, National University Hospital, Singapore, Singapore

**Keywords:** Frosted branch angiitis, ATT, Mantoux, QuantiFERON, COTS

## Abstract

**Aim:**

To illustrate the application of the Collaborative Ocular Tuberculosis Study (COTS) calculator in guiding the management of a case of frosted branch angiitis (FBA) secondary to presumed intraocular tuberculosis (TB).

**Methods:**

Retrospective case report.

**Results:**

A 33-year-old Indonesian female, with no significant past medical history except for previous exposure to pulmonary tuberculosis in childhood, presented with acute blurring of vision, redness, and pain in the right eye for one day. On eye examination, her right eye exhibited a visual acuity of counting fingers at 2 m and a normal IOP of 18 mmHg. The anterior chamber of the right eye showed 3 + cells, while the fundus revealed retinal vasculitis in a classic FBA-like pattern and macular edema. The left eye had a visual acuity of 6/9, with normal findings on fundus examination. Fundus fluorescein angiogram showed predominantly venular leakage with optic disc leakage. Systemic evaluation showed a positive QuantiFERON-TB Gold test and a weakly positive Mantoux test (6 mm with 1 TU), with no radiological evidence of active pulmonary TB on chest X-ray. Based on clinical assessment, COTS calculator inputs, and multidisciplinary discussion, anti-tubercular therapy (ATT) was deferred. The patient was treated with systemic corticosteroids, resulting in resolution of macular edema and recovery of visual acuity to 6/6.7 in both eyes at 3 months. Within a span of two months with steroidal treatment alone, she experienced complete resolution of cystic macular edema and achieved a BCVA of 6/6.7 in both eyes.

**Conclusion:**

The COTS calculator may support clinical decision-making in selected cases of presumed intraocular TB; however, management should be individualized, supported by multidisciplinary input, and accompanied by close long-term follow-up.

## Introduction

Intraocular tuberculosis (TB) caused by Mycobacterium tuberculosis (MTB) may present with various features, including granulomatous anterior uveitis, intermediate uveitis, retinal vasculitis, choroiditis, and cystoid macular edema. In addition to clinical signs, diagnosis should be supported by positive corroborative investigations indicative of tuberculosis [1]. Extraocular TB manifestations and differentials must be considered before initiating anti-tubercular treatment (ATT). This process involves a series of blood tests and chest radiography [2]. The **COTS** study remains a large-scale, multi-centric retrospective clinical study that provides standardized global recommendations and guidance for the management of presumed and definitive intraocular tuberculosis [3]. The study also developed an online tool called the COTS calculator based on consensus from various experts on specific case scenarios. We report a case of frosted branch angiitis (FBA)-like retinal vasculitis managed with corticosteroids without ATT, guided by clinical assessment, COTS inputs, and multidisciplinary evaluation.

## Case report

A 33-years-old Indonesian female domestic helper presented with a one-day history of blurred vision in her right eye, accompanied by redness and pain. She had no significant past medical history except previous exposure to pulmonary tuberculosis in her childhood details of which were not available as contact happened in overseas. Her best-corrected visual acuity was counting fingers at 2 m in the right eye and 6/9 in the left eye. Intraocular pressures were 18 mmHg in the right eye and 19 mmHg in the left eye. Her right eye exhibited a grade 2 relative afferent pupillary defect.

**Fig. 1:**
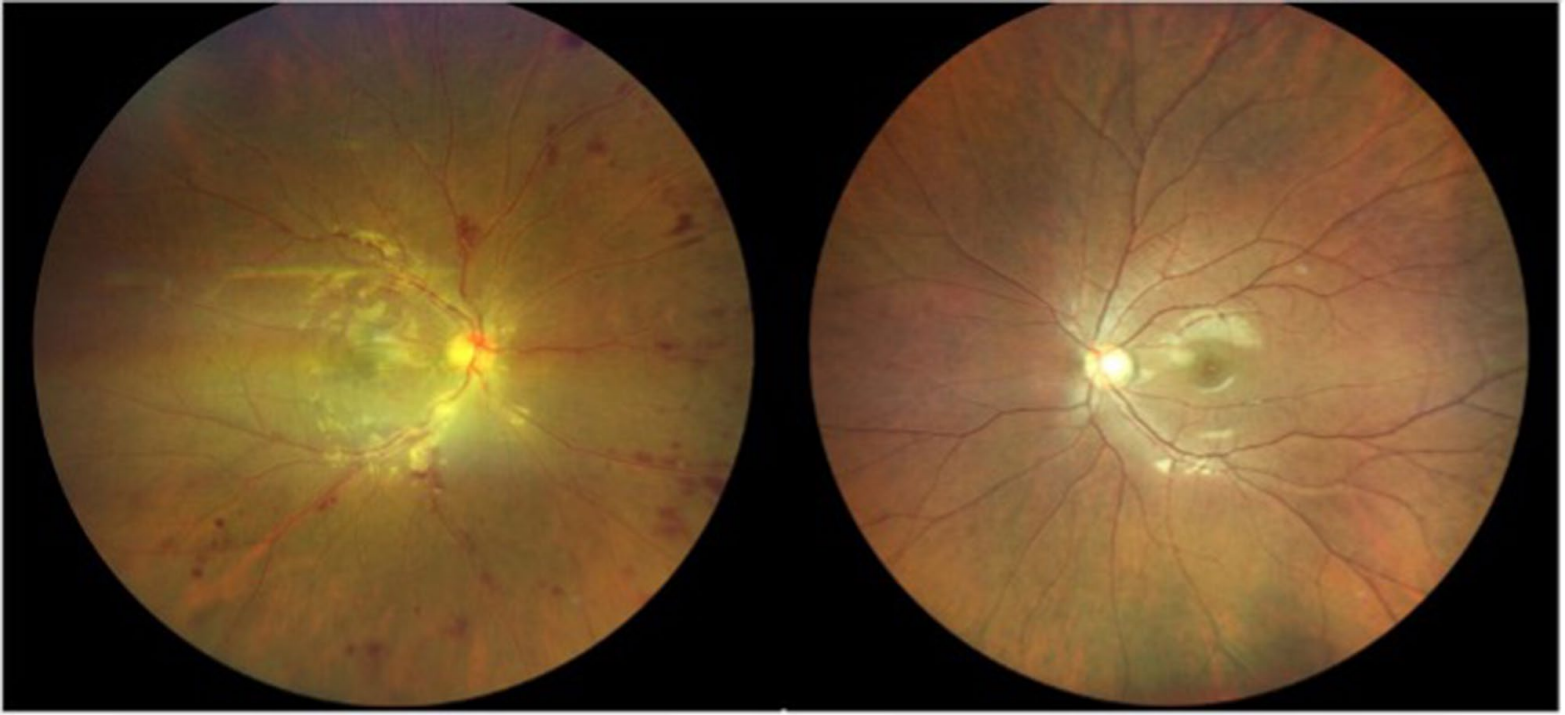
Fundus photo of right eye showing severe retinal vasculitis in frosted branch tree like pattern in right eye and normal fundus examination in the left eye

**Fig. 2:**
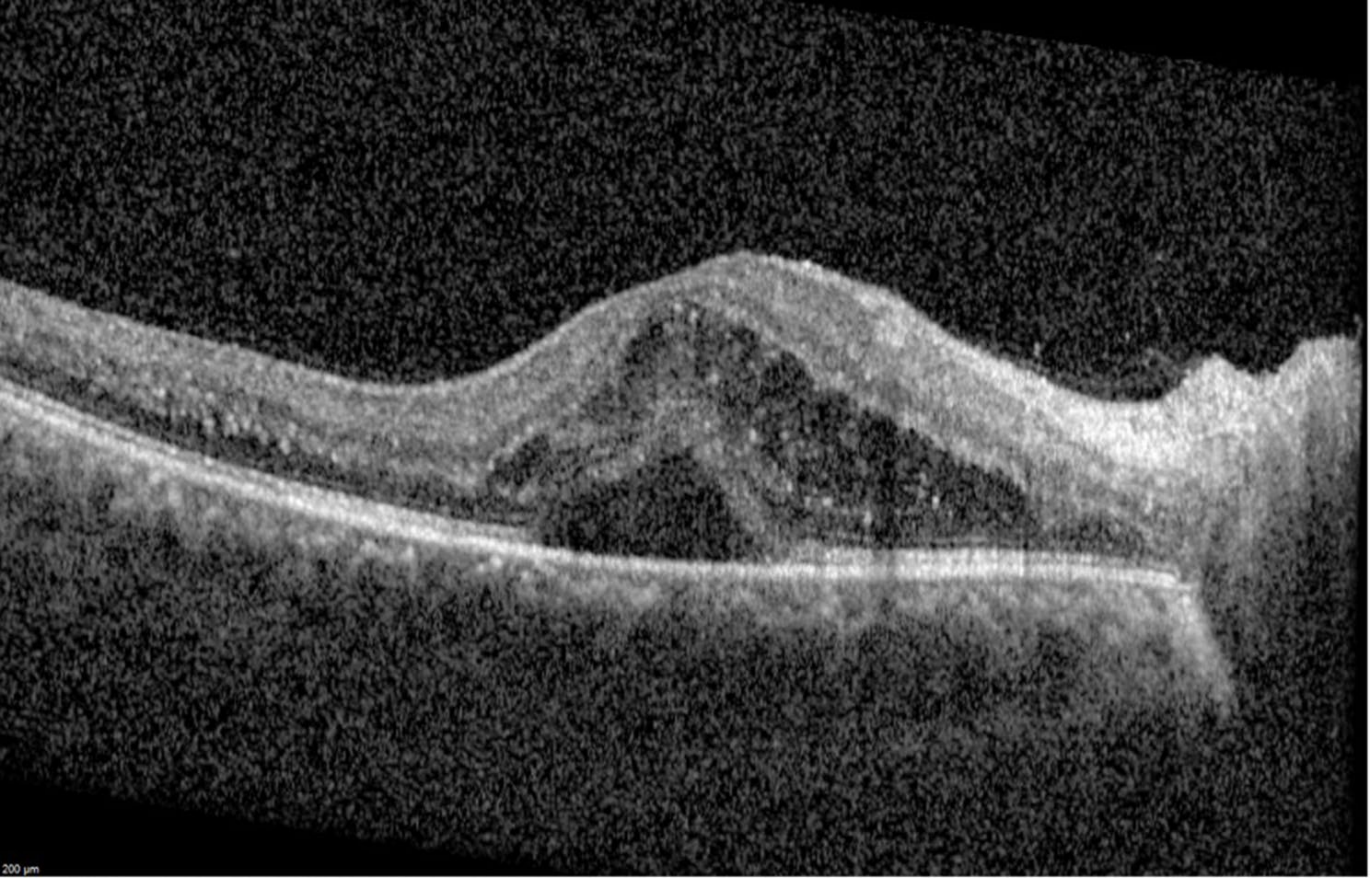
OCT macula of right eye showing cystoid macular edema in right eye

**Fig. 3:**
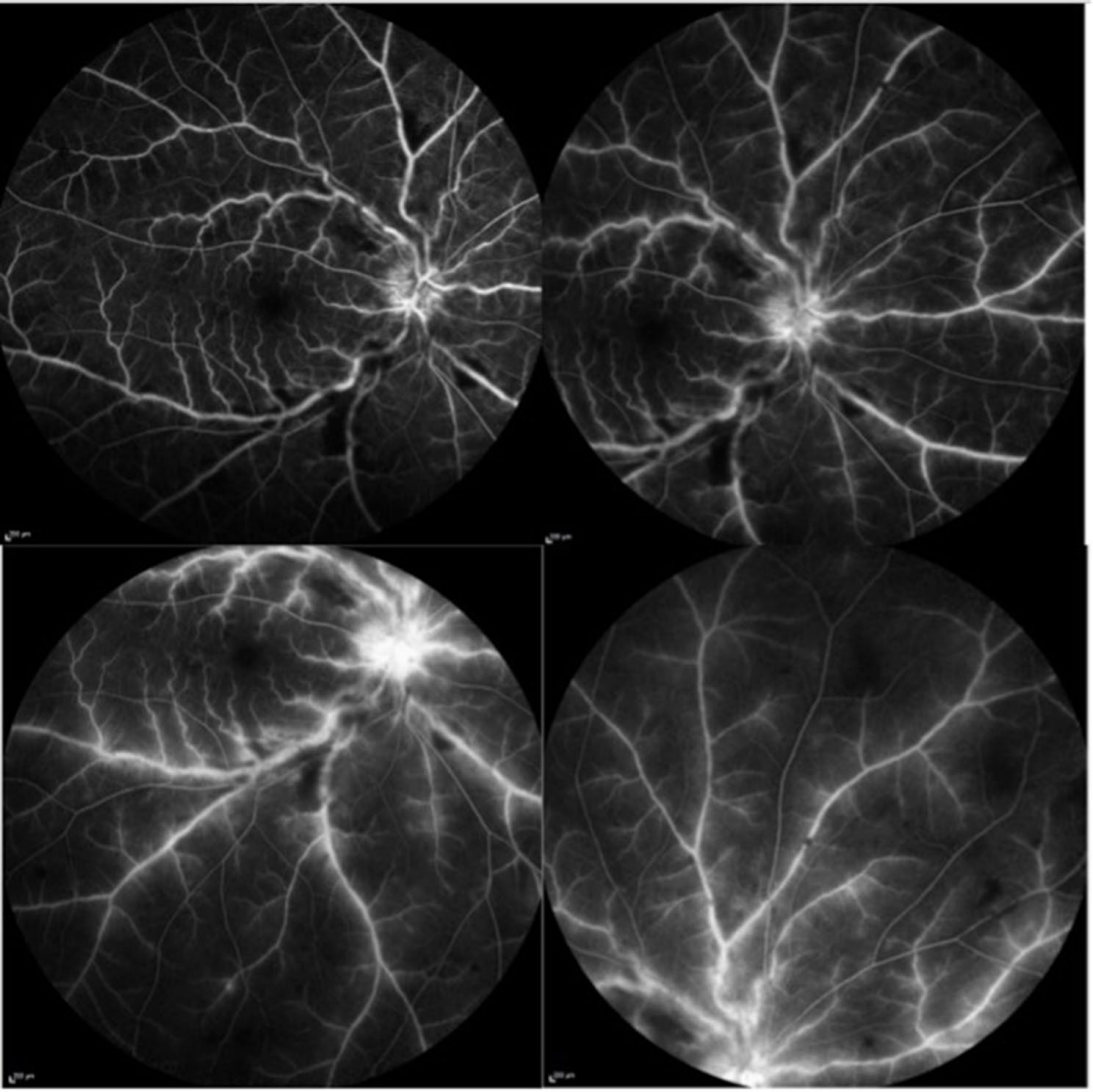
Fundus Fluorescein angiography of the right eye at 1 min, 2-minute, 5.5 min and 8 min interval showing vasculitis involving predominantly venules with disc leakage

The anterior segment examination of the right eye revealed conjunctival injection and grade 3 + anterior chamber cells. There was no clinically significant vitritis; however, a small number of vitreous cells were noted on OCT. Fundus examination revealed macular edema and retinal vasculitis in an FBA-like pattern predominantly involving the posterior pole (Fig. 1). OCT confirmed cystoid macular edema with foveal detachment **(**Fig. 2). FFA demonstrated predominantly venular leakage with optic disc leakage **(**Fig. 3). Systemic workup showed a positive QuantiFERON-TB Gold test the values are NIL Tube 0.580 Tube1- 3.710 Tube 2-4.690 MIT-NIL tube 9.570 respectively. Mantoux testing with 1 Tuberculin unit (TU) demonstrated 6 mm induration after 48 h. Serology for HIV, hepatitis B and C, and syphilis was negative. Autoimmune markers (ANA, anti-dsDNA, ANCA) were negative. Full blood count and renal panel were within normal limits. Chest X-ray showed no evidence of active or prior pulmonary TB. The patient declined anterior chamber tap for microbiological analysis.

Based on clinical findings and investigations, presumed intraocular TB was considered. COTS calculator inputs suggested a low likelihood of requiring ATT in this scenario. After multidisciplinary discussion with the infectious disease team, a decision was made to treat with corticosteroids and observe, with ATT reserved for clinical deterioration or recurrence.

**Fig. 4:**
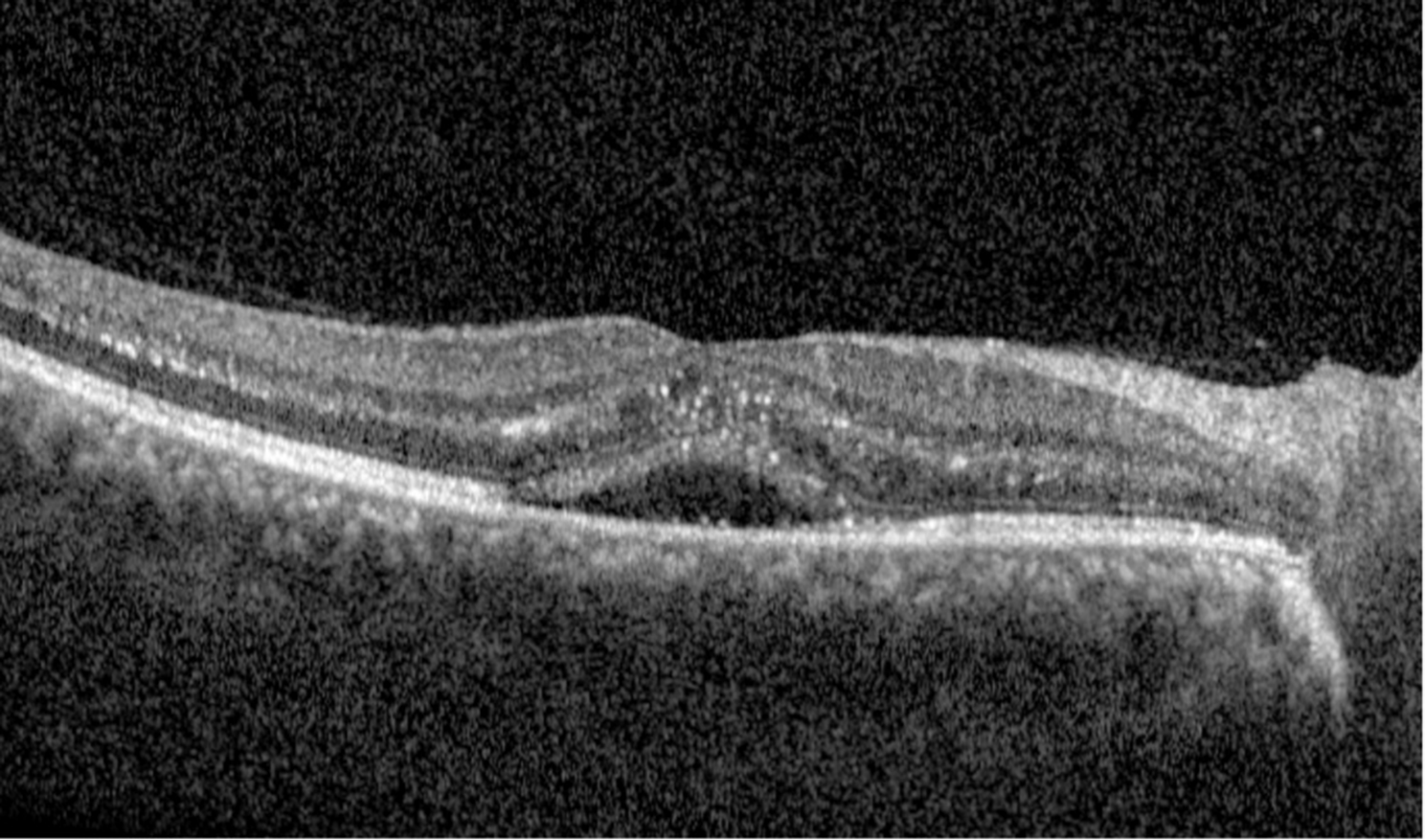
OCT Macula of right eye showing improvement of cystoid macular oedema

**Fig. 5:**
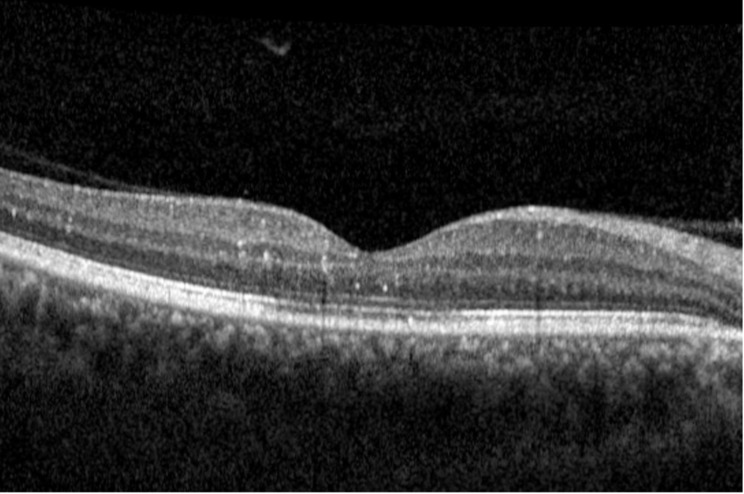
OCT Macula of right eye showing complete resolution of cystoid macular oedema in the right eye and fundus photo right eye showing resolution of vasculitis

The patient was started on topical prednisolone acetate 1% every 3 h in the right eye and oral prednisolone 40 mg/day. At 4 days, vision improved to 6/30 (right), with reduction in macular edema on OCT (Fig. 4). Oral prednisolone was tapered (30 mg for 5 days, 20 mg for 5 days, 15 mg for 5 days, 12.5 mg for 5 days, then 10 mg maintenance), and topical therapy was discontinued. At 3 months, BCVA improved to 6/6.7 in both eyes. Fundus examination showed resolving vasculitis with trace preretinal hemorrhage and OCT demonstrated complete resolution of cystoid macular edema (Fig. 5). Follow-up FFA was not performed.

Despite the response to oral prednisolone, an infectious disease referral was also made in view of her positive QuantiFERON-TB Gold (QFT). However, the infectious disease team also concurred, recommending treatment as latent tuberculosis, with active anti-tubercular medication initiated only if ocular symptoms deteriorated or if there was a recurrence of uveitis. Hence, anti-tubercular therapy (ATT) was not initiated.

## Discussion

Frosted branch angiitis is one of the morphological forms of retinal vasculitis that can cause vision-threatening complications in immunosuppressed patients, most commonly due to cytomegalovirus (CMV) [4]. Various other causes of FBA have been reported in the literature, including intraocular tuberculosis and certain autoimmune conditions. Accurate identification of the underlying cause is essential as management differs significantly. ***Gupta et al.*** classified intraocular TB into presumed, probable, and possible categories based on clinical findings, as well as microbiological, immunological, and radiological tests [5]. The **COTS-1**, a retrospective multinational cohort study, was the largest data set on ocular TB and combined data from 25 leading international eye care centers, guided by renowned uveitis specialists from around the world, including a total of 945 patients diagnosed with ocular TB [1]. The diagnostic criteria based on COTS-1 requires clinical signs of TB uveitis and exclusion of other uveitic entities, with investigations suggestive of mycobacteria [1]. Management of active intraocular tuberculosis with ATT also reduces the rate of recurrences in future [4–6]. The COTS calculator uses a two-step Delphi method involving 81 experts who evaluated 468 clinical scenarios [7].

In our index case, inputs into the COTS calculator suggested a lower likelihood of requiring ATT; however, we acknowledge that scoring may vary depending on interpretation of individual parameters. Importantly, the decision to defer ATT was not based solely on the COTS calculator but was made in conjunction with infectious disease specialists, who recommended close monitoring and management as latent TB. The patient demonstrated rapid improvement with systemic corticosteroids, suggesting a predominantly inflammatory process at presentation. While short-term response to corticosteroids alone has been described in cases of presumed ocular TB, this approach should be used cautiously, as it does not address potential underlying infection and may mask disease activity.

The presence of a relative afferent pupillary defect and optic disc leakage indicated significant posterior segment inflammation, supporting the use of systemic corticosteroids. The patient responded well to a moderate-dose regimen with gradual tapering. Long-term follow-up (> 1 year) is essential, as recurrence is well documented in ocular TB. The 3-month follow-up in this case is relatively short and represents a limitation. Continued surveillance is necessary to detect relapse and reassess the need for ATT.

Alternative diagnoses, including sarcoidosis and other infectious or autoimmune causes of retinal vasculitis, were considered. Negative laboratory investigations reduced the likelihood of these conditions. Additional limitations include the use of chest X-ray rather than contrast-enhanced CT, which may miss subtle pulmonary findings, the absence of follow-up FFA and being a single case report, additional case series are required.

## Conclusion

The **COTS** calculator can serve as a useful adjunct in evaluating atypical retinal vasculitis where intraocular TB is suspected. However, it should not replace comprehensive clinical assessment and multidisciplinary evaluation. In selected patients with low clinical suspicion of active TB, a carefully monitored trial of corticosteroid therapy may be considered. Long-term follow-up is essential, and the need for ATT should be reassessed if there is any clinical deterioration or recurrence. Our case highlights its importance and recommends for future use.

## Data Availability

No datasets were generated or analysed during the current study.

## Abbrevations

COTS: Collaborative ocular tuberculosis study
ATT: Anti-tubercular therapy
TB: Tuberculosis
OCT: Optical coherence tomography
FBA: Frosted branch angiitis
